# Correlation of narrow band imaging vascular patterns with immunohistological microvessel density in vocal fold lesions

**DOI:** 10.1016/j.bjorl.2019.07.009

**Published:** 2019-09-17

**Authors:** Anna Rzepakowska, Michał Żurek, Jakub Grzybowski, Paweł Pihowicz, Barbara Górnicka, Ewa Osuch-Wójcikiewicz, Kazimierz Niemczyk

**Affiliations:** a Medical University of Warsaw, Otolaryngology Department, Warszawa, Poland; b Medical University of Warsaw, Department of Pathology, Warszawa, Poland

**Keywords:** Intraepithelial lesion, Dysplasia, Early glottis cancer, Microvessel density, Narrow band imaging

## Abstract

**Introduction:**

The microarchitecture of the mucosal and submucosal vessels is crucial for diagnosis of vocal fold lesions. Neo-angiogenesis is a confirmed biological parameter that implicates progression and metastasis in laryngeal cancer.

**Objective:**

This study investigates the correlation between vascular pattern classifications by narrow band imaging and immunohistological microvessel density in different types of intraepithelial vocal fold lesions.

**Methods:**

Analysis of immunohistological microvessel density using CD31 and CD34 antibodies was performed in 77 lesions including: 20 non-dysplastic lesions, 20 with low-grade dysplasia, 17 with high-grade dysplasia and 20 invasive cancers. The evaluation of vascular patterns with narrow band imaging according to the Ni classification and European Laryngological Society guidelines was performed prior to surgical resection.

**Results:**

The mean value of CD31 microvessel density was the highest for Ni Type IV lesions (20.55), whereas for the longitudinal and perpendicular patterns according to the European Laryngological Society classification it was 12.50 and 19.45 respectively. The highest mean value of microvessel density with CD 34 was identified in Ni Type Va (35.43) lesions and in the longitudinal and perpendicular patterns according to the European Laryngological Society classification was 15.12 and 30.40 respectively.

**Conclusions:**

The microvascular morphological changes of intraepithelial laryngeal lesions observed under narrow band imaging endoscopy are positively correlated with angiogenesis indexes of immunohistological evaluation.

## Introduction

Technological advances in endoscopic imaging, including magnification endoscopy and Narrow Band Imaging (NBI), have provided laryngologists with clinical tools for identification of laryngeal intraepithelial lesions with dysplasia or malignant potential with diagnostic accuracy closely corresponding to histopathology evaluation. NBI enables detailed evaluation of morphology and mucosal vessels without the need for staining agents. The microarchitecture of the mucosal and submucosal vessels is crucial for diagnosis. Neo-angiogenesis is a confirmed biological parameter that implicates progression and metastasis in laryngeal cancer.^1^ Research on progression from intraepithelial dysplasia to early invasive cancer in the uterine cervix and esophagus also showed the concurrent increase in microvascular density in the epithelium with the grade of atypia on histopathology.^2^, ^3^

The first results confirming the effectiveness of the NBI method for differentiation of lesions in the larynx appeared in the English literature in 2010.^4^ Piazza et al. on the basis of a prospective analysis of 279 patients defined oncologically suspicious features of laryngeal mucosa identified by NBI: “well-demarcated brownish area with thick dark spots and/or winding vessels, and afferent hypertrophic vessel branching out in small vascular loops in the context of the lesion”. At that time, Ni verified, presented and published a proposal for a classification of NBI types of mucosal vascularization patterns in laryngeal lesions, and verified that it is an effective assessment tool.^5^

The five types of vascular patterns from I to V (Type V with subtypes: Va, Vb, Vc) were created, basing on the previously proposed scale for the assessment of NBI changes in the esophagus by Inoue et al.^6^ For the next 5 years, the Ni classification was the only tool used in research on the diagnostic effectiveness of NBI in the larynx. The system by Ni et al. is simple, however the distinction of Type I (normal in size) and II (enlarged in size and density) for longitudinal vessels is not useful in the differential diagnosis of pre-cancerous lesions and Type III (leukoplakia covering vessels) is uninformative. However the following types identify perpendicular vascular loops in the epithelium and seem to describe changes in microvessel architecture with progression of underlying histopathology: Type IV (loops of small size, regular shape, and low density), Va (irregular, twisted, and swirled loop shapes, larger in size and of higher density), Vb (earthworm-shaped capillary loop remnants) and Vc (necrotic tissue with remnants of capillary loops). According to Ni et al. NBI Type IV is believed to correspond with low-grade dysplasia, Type Va with high-grade dysplasia and carcinoma in situ and Type Vb and Vc with invasive cancer. High grade dysplasia within Type IV lesions and infiltrating cancer in Type Va is possible, therefore a straightforward assignment of NBI types to a grade of intraepithelial dysplasia or invasive lesion is not possible. In 2016, the committee on endoscopic laryngology imaging of the European Laryngological Society (ELS) proposed the descriptive guideline of vascular changes in vocal fold lesions.^7^ The extensive descriptive version of the ELS system can seem quite inconvenient for clinical application. Nevertheless for learning purposes and acquiring experience it seems to be more comprehensive. For daily practice the general distinction of the ELS system, concerning the direction of mucosal vessels (longitudinal or perpendicular), is crucial for differentiation between benign and suspicions lesions of dysplasia or invasive cancer.^7^, ^8^

Currently NBI is accepted and commonly used worldwide for accurate diagnosis and follow-up in patients with laryngeal lesions.^9^ It is difficult to create a perfect classification system, but the coexistence of two rating scales may hinder uniform assessment and comparison of results.

Furthermore we did not find any studies that investigated the correlation between NBI vascular patterns in laryngeal intraepithelial lesions and immunohistologically-assessed microvessel density.

Therefore this study was designed to identify the relationship between the two classification systems of NBI with immunohistologically evaluated vascular density within vocal fold lesions.

## Methods

The protocol of the study was approved by the local Ethical Review Board (KB/83/2017). All procedures performed in the study were in accordance with the 1964 Helsinki declaration and its later amendments or comparable ethical standards. Informed consent was obtained from all individual participants included in the study.

The study was performed in 2018 by retrospectively retrieving the archived medical records and paraffin-embedded tissue blocks of patients treated with laryngeal microsurgery in our department due to suspicious hypertrophic lesions of the VFs. All participants had NBI evaluation of the lesion prior to resection.

The study design assumed inclusion of patients with different histological grades of VFs pathologies: non-dysplastic lesions (stromal edema, inflammatory changes), low-grade dysplasia, high-grade dysplasia (with carcinoma in situ) and invasive cancer in a comparable amount. The patients were consecutively enrolled in each group. We excluded patients who: had a history of previous laryngeal microsurgery or biopsy, radiotherapy in the neck area or a history of immunodeficiency syndrome and systemic chemotherapy and those with inadequate amount of tissue specimen in paraffin-embedded blocks for reliably performed immunohistochemistry procedure. Finally we included a total number of 77 patients (20 with non-dysplastic lesions, 20 with low grade dysplasia, 17 with high grade dysplasia, 20 with invasive cancers) with sufficient tissue material for further staining procedures. The specimens obtained during microsurgical resection or tissue biopsy from VF lesions were checked again by two histopathologists, blinded to the initial results, in order to confirm the diagnosis according to World Health Organization classification from 2017.^10^ In cases with divergent opinions consensus was achieved before inclusion for further analysis.

### Narrow band imaging evaluation

The entire length of the preoperative NBI examination recordings with the Visera Elite OTV-S190 video system and endoscopic nasopharyngolaryngoscopy ‒ VH videoendoscope (Olympus Medical Systems, Volketswil, Switzerland) were analyzed, and used to evaluate the vascular patterns within the lesions according to the Ni classification and the ELS staging system by two specialists experienced with the method and blinded to the patients’ data and histopathology result. The inter-observer agreement for Ni and ELS classification was 88% and 99%, respectively. Consensus was established for cases with different opinions. The NBI examinations had been performed with the patient seated after topical anesthesia of nasal cavity and if necessary also of the throat with lidocaine spray 0–3 days before surgical resection. The examination was started using white light, then switched to the standard NBI mode and finally to the magnified NBI mode.

### Immunohistochemistry procedure

CD31 and CD34 antibodies that are most commonly selected in the studies assessing microvascular density on Immunohistochemistry (ICH) staining were chosen for our investigation. CD31 is detected in mature endothelial cells ranging from nonlumen to lumen-forming cells.^11^ CD34 is a transmembranous sialoprotein detected with anti CD34 antibodies both in precursors of endothelial cells and in differentiated endothelial cells.^12^ The limited number of possible IHC procedures in examination of vocal fold specimens depends on the small amount of material obtained during the laryngeal microsurgery and the accompanying thermal changes of the tissue margins, due to the laser resection. 3–5 μm thick sections were obtained from 77 formalin fixed paraffin embedded tissue blocks with a microtome (HM 340E Electronic Rotary Microtome, Thermo Shandon). All sections were stained in na automatic tissue processor (ASP 6026, Leica, USA) with hematoxylin and eosin (H&E). Corresponding sections were stained with anti-CD31 antibody (clone: JC70A; DAKO/Agilent, USA), anti-CD34 antibody (clone: QBEnd10; DAKO/Agilent, USA). EnVision FLEX, High pH (Link) (DAKO/Agilent, USA) and Autostainer Link 48 (DAKO/Agilent, USA) were used to perform immunostaining.

In brief, the tissue sections were deparaffinized by incubation in a wash buffer, pH 9.0, at 98 °C for 20 min in PT Link station (DAKO/Agilent, USA). After 10 min of incubation in the wash buffer at room temperature the endogenous peroxidase activity was blocked by immersion in a 3% hydrogen peroxide solution for 10 min. The slides were then incubated for 20 min with the primary antibodies, which was followed by addition of the secondary antibody. In case of anti-cluster of differentiation (CD) 31 antibody, the reaction was strengthened by adding Mouse LINKER according to the producer’s recommendations. Visualization was achieved by application of 3.3′-diaminobenzidine. Finally, cell nuclei were counterstained with hematoxylin. The tissue sections were dehydrated with graded concentrations of ethanol, cleared with xylene and covered with tape cover slipper (Klinipath, The Netherlands).

Evaluation of immunostained sections was carried out by two histopathologists with light microscopy and the results were averaged.

### Evaluation of microvessels density

Firstly, for each slide the regions with highest vessel density (hotspots) were identified at low magnification (×40), then the average count of CD31-positive and CD34-positive vessels in three or five hotspots respectively was calculated at high magnification (×200). Only continuous membranous staining was considered positive. Any large microvessel with a lumen or any single, separated endothelial cell was counted as 1. The vessels were counted within the epithelium and at the epithelium/stroma interface.

**Figure 1:**
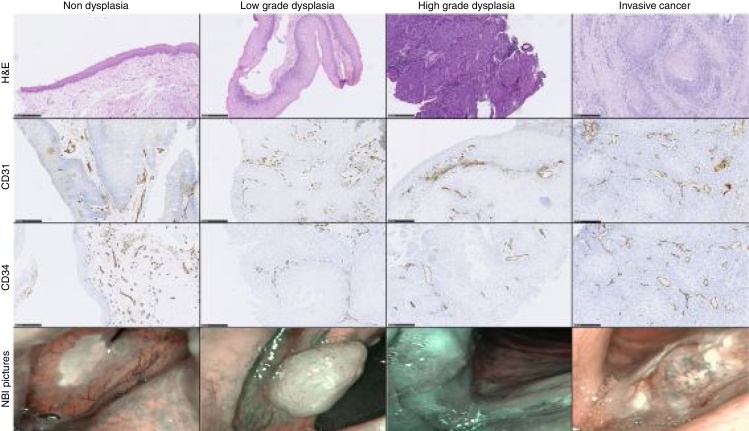
Examples of H&E.

Fig. 1 presents examples of H&E stained sections and immunostaining for both CD31 and CD34 in each type of intraepithelial VF lesion at ×100 magnification with respective NBI pictures.

### Statistical analysis

Parameters were evaluated using SPSS 18.0 and Statistica 13. A *p*-value lower than 0.05 was considered statistically significant for all analyses. The interrater agreement in NBI assessment was calculated as a percentage agreement. The ANOVA, nonparametric Kruskal-Wallis and Mann-Whitney U tests were used to compare differences between NBI types and expression of CD31 and CD34. The ANOVA and Kruskal-Wallis test were used to prove, if there are statistically significant differences for all analyzed vascular patterns. The Ni classification types of NBI were divided into two groups: Types I—IV to benign group and Types Va‒Vc to malignant group. With Mann-Whitney U test the mean MVD of each type was compared to average value of MVD of opposite group. The mean MVD between longitudinal and perpendicular pattern (according to ELS classification) was also compared using Mann-Whitney U test. Box plots were used for graphical presentation of aquired results. Correlations between both methods evaluating microvessels density (MVD CD31 and MVD CD34) with NBI and ELS vascular patterns were calculated with the Spearman Rank Correlation test.

## Results

In each of the 77 lesions immunohistochemical analysis of MVD with CD31 and CD34 antibodies as well as assessment of the vascular patterns in NBI endoscopy was performed. The non-dysplastic lesions in 19 cases presented Ni Type II and in 1 case NI Type I. Within this group of lesions all vessels were longitudinal. The 20 lesions with low grade dysplasia presented: 2 ‒ Ni Type II, 4 ‒ Ni Type III, 12 ‒ Ni Type IV and 2 ‒ Ni Type Va vascular patterns. Longitudinal vessels were identified in 6 lesions of low-grade dysplasia, and perpendicular vessels were present in 14. Among the lesions with high grade dysplasia the following NBI patterns were identified: 1 – Ni Type III, 1 – Ni Type IV, 6 – Ni Type Va, 6 ‒ Ni Type Vb and 3 ‒ Ni Type Vc. Longitudinal vessels were present in 1 case, the remaining 16 lesions had perpendicular vessels. The invasive cancers demonstrated: 2 ‒ Ni Type Va, 8 ‒ Ni Type Vb and 10 ‒ Ni Type Vc vascular patterns. All 20 cases of invasive cancers had perpendicular vessels within the lesion. Table 1 presents demographical data and detailed NBI vascular patterns according to the Ni and ELS classification for individual groups according to histopathological diagnosis.

**Table 1:** Demographical data and the NBI vascular patterns according to the Ni classification and European Laryngological Society (ELS) staging for individual histopathological lesion types.

	Number of lesions	Age mean ± SD	NBI Type I	NBI Type II	NBI Type III	NBI Type IV	NBI Type Va	NBI Type Vb	NBI Type Vc	ELS longitudinal	ELS perpendicular
Non-dysplastic	20	55.15 ± 12.38	1	19	‒	‒	‒	‒	‒	20	‒
Low-grade dysplasia	20	61.55 ± 10.66	‒	2	4	12	2	‒	‒	6	14
High-grade dysplasia	17	64.94 ± 11.25	‒	‒	1	1	6	6	3	1	16
Invasive cancer	20	66.65 ± 9.31	‒	‒	‒	‒	2	8	10	‒	20
All lesions	77	61.96 ± 11.62	1	21	5	13	10	14	13	27	50

Microvascular density assessment using CD31 expression (CD31 MVD) in endothelial cells revealed statistically significant differences in microvessel count between NI classifications and between ELS classifications (*p*-value of Kruskal Wallis test for Ni classification and Mann-Whitney U test for ELS classification 0.016 and 0.002 respectively). The mean value of CD31 MVD progressively increased from Type II (12.63) up to the highest value for Type IV (20.55). The greatest decrease in CD31 MVD was seen for Type Va (17.67) but rose again in Types Vb and Vc (19.67 and 19.92 respectively).

**Figure 2:**
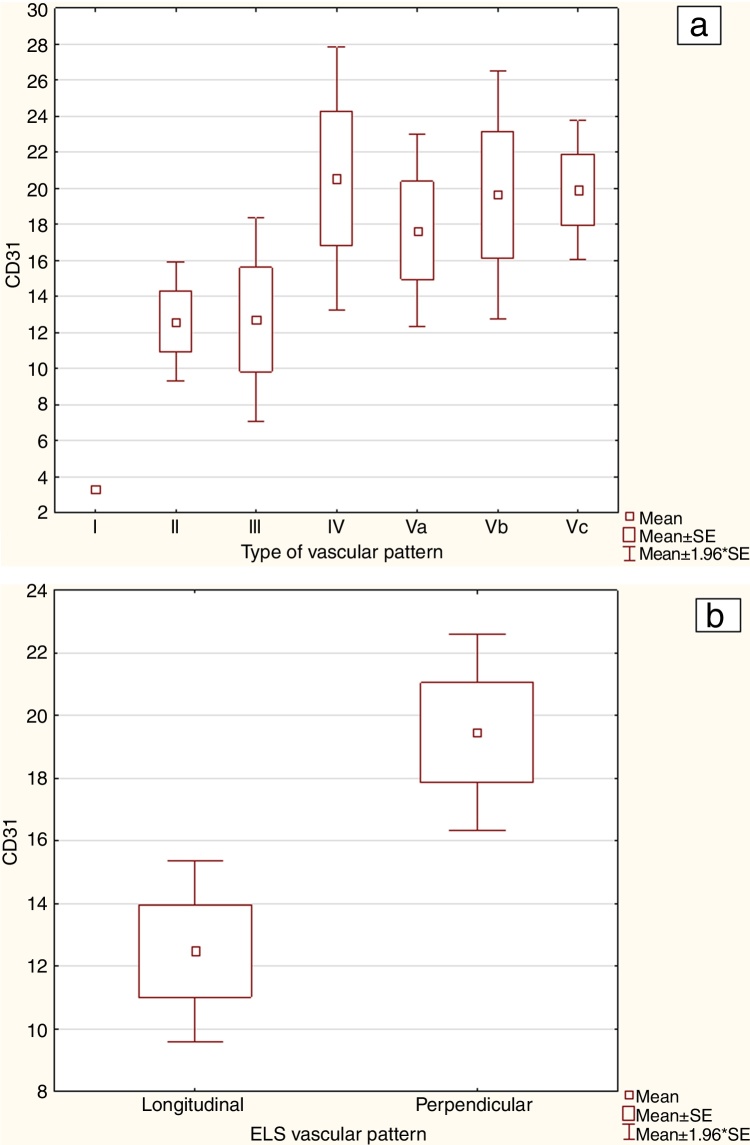
Graphical statistics for CD31 MVD (2A) and ELS classifications (2B).

The CD31 MVD in lesions with a longitudinal vessel pattern according to the ELS classification was 12.5 compared to 19.45 for those with perpendicular vessels. The detailed statistics of CD31 MVD in vascular patterns with Ni and ELS classification are presented in Table 2. Fig. 2 presents graphical statistics for CD31 MVD in NBI types according to Ni (Fig. 2A) and ELS classifications (Fig. 2B).

**Table 2:** The mean microvessel density (MVD) with CD31 in individual vascular pattern types according to the Ni and European Laryngological Society (ELS) classifications.

NBI classification	Number of analyzed lesions	Mean CD31 MVD	Range of CD31 MVD	Standard deviation (±SD) CD31 MVD	Standard error CD31 MVD	95% CI CD31 MVD	*p*-value
Type I	1	3.33	‒	‒	‒	‒	‒
Type II	21	12.63	3.00‒33.33	7.87	1.68	9.14‒16.11	0.04^a^
Type III	5	12.73	5.33‒ 21.00	6.44	2.88	4.74‒ 20.73	0.115^a^
Type IV	13	20.55	8.67‒ 56.00	13.95	3.73	12.49‒28.60	0.842^a^
Type Va	10	17.67	5.67‒ 26.67	7.19	2.72	11.02‒24.32	0.198^a^
Type Vb	14	19.67	6.00‒53.67	13.59	3.51	12.14‒27.19	0.16^a^
Type Vc	13	19.92	9.00‒34.67	7.11	1.97	15.63‒24.22	0.013^a^
Total	77	17.01	3.00‒56.00	10.63	1.21	14.60‒19.43	0.016^b^
ELS longitudinal pattern	27	12.50	3.00‒33.33	7.65	1.47	9.47‒15.52	0.02^a^
ELS perpendicular pattern	50	19.45	5.67‒56.00	11.27	1.59	16.25-22.65	

95% CI, Confidence Interval 95%.

a *p*-value of Mann-Whitney U test.

b *p*-value of Kruskal-Wallis test.

Microvessel density assessment using CD34 expression (CD34 MVD) also revealed statistically significant differences in the mean microvessel count between NI classification and between ELS classification (*p*-value of ANOVA test for Ni classification and Mann-Whitney U test for ELS classification both <0.001).

For CD34 MVD the highest mean value was identified in lesions with Type Va (35.43) compared to Type IV (25.94), Type Vb (30.48) and Vc (34.45).

**Figure 3:**
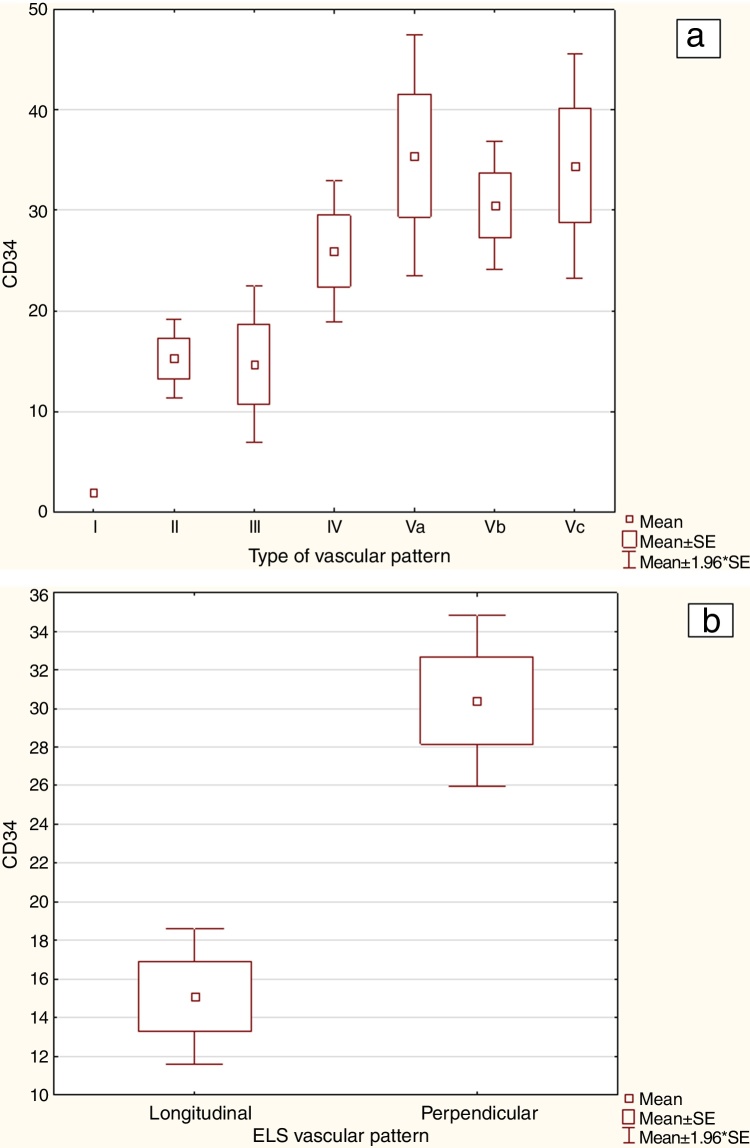
Graphical statistics for CD34 MVD (3A) and ELS classifications (3B).

The CD34 MVD in lesions with a longitudinal pattern according to the ELS classification was 15.12 compared to 30.40 for those with perpendicular vessels. The detailed statistics of CD34 MVD for each vascular pattern type according to the Ni and ELS classifications are presented in Table 3. Fig. 3 present’s graphical statistics for CD34 MVD in NBI types according to Ni (Fig. 3A) and ELS classifications (Fig. 3B).

**Table 3:** The mean microvessel density (MVD) with CD34 in individual vascular pattern types according to the Ni and European Laryngological Society (ELS) classifications.

NBI classification	Number of analyzed lesions	Mean CD34 MVD	Range of CD34 MVD	Standard deviation (±SD) CD34 MVD	Standard error CD34 MVD	95% CI CD34 MVD	*p*-value
Type I	1	2.00	‒	‒	‒	‒	‒
Type II	21	15.30	3.40‒3180	9.47	2.02	11.10‒19.50	<0.001^a^
Type III	5	14.72	2.80‒25.40	8.82	3.97	3.69‒25.75	0.08^a^
Type IV	13	25.94	7.60‒49.40	13.39	3.58	18.20‒33.68	0.153^a^
Type Va	10	35.43	17.00‒57.40	16.14	6.10	20.50‒50.36	0.01^a^
Type Vb	14	30.48	6.80‒56.00	12.49	3.27	23.56‒37.40	0.02^a^
Type Vc	13	34.45	10.00‒92.60	20.54	5.70	22.04‒46.86	0.03^a^
Total	77	25.04	2.00‒92.60	15.75	1.79	21.47‒28.62	<0.001^b^
ELS longitudinal pattern	27	15.12	2.00‒31.80	9.27	1.79	11.44‒18.90	<0.001^a^
ELS perpendicular pattern	50	30.40	4.00‒92.60	15.98	2.26	25.86‒ 39.44	

95% CI, Confidence Interval 95%.

a *p*-value of Mann-Whitney U test.

b *p*-value of ANOVA.

The correlation (Spearman’s test) between NBI Ni classification and CD31 MVD and CD34 MVD revealed a rho value of 0.333 and 0.521 respectively (*p*-value 0.003 and <0.001 respectively). The correlation between ELS classification and CD31 MVD and CD34 MVD revealed a rho value of 0.356 and 0.506 respectively (*p*-value 0.02 and <0.001 respectively).

## Discussion

This study is the first report in the literature that utilized pathology techniques to assess the correlation between MVD and disease progression of non-dysplastic lesions, lesions with low-grade dysplasia, lesions with high-grade dysplasia and invasive cancer of the vocal folds evaluated with NBI endoscopy. We investigated the vocal fold mucosal vascular density immunohistochemically using CD31 and CD34 antibodies and demonstrated good agreement with both Ni and ELS classifications of microvascular patterns in NBI. We also showed a significant stepwise increase in MVD from NBI vascular patterns typical for nondysplastic VF lesions (Type I or II according to Ni classification or longitudinal vessels in ELS classification), to NBI models characteristic for dysplastic and invasive cancer (Type IV and V or perpendicular vessels). The NBI technique was designed to highlight the vascularization of the mucosa through application of special light filters, which isolate the wave lengths that are absorbed by hemoglobin. The high accuracy of this endoscopic method has been confirmed in the differential diagnosis of laryngeal lesions in many studies.^4^, ^5^, ^8^, ^13^, ^14^, ^15^, ^16^, ^17^, ^18^, ^19^ Most of the studies have used the Ni classification due to its accuracy, specificity and sensitivity. However, the main limitation of NBI is the horizontal visualization of mucosal vessels without direct examination of the depth. The Ni classification of vascular patterns was designed to reflect the stepwise progression of perpendicular vessels from being small in size and of low density described as Type IV in low-grade dysplasia, to larger in size, distorted and of higher density for Type Va in high-grade dysplasia and carcinoma in situ and interruption of vessels and foci of necrosis in Type Vb and Vc, which is typical for invasive cancer. This classification of laryngeal mucosal vessels by Ni was modeled on the classification of the Intrapapillary Capillary Loop (IPCL) pattern introduced by Inoue et al. for assessment of superficial esophageal lesions.^7^ There were some doubts as to whether the differences in the histological structure of the vocal folds and esophagus epithelium do not cause discrepancies in the assessment of IPCL types within the larynx. The classification proposed by ELS was created for more a practical approach with a dichotomous distinction of benign (longitudinal vessels) and premalignant or malignant (perpendicular vessels) lesions.^7^ Not only did we not find any studies concerning the feasibility of visualizing angiogenesis by NBI in laryngeal intraepithelial lesions by comparing to immunohistochemical evaluation of microvascular density, a lack of investigations exists concerning neovascular progression in carcinogenesis of laryngeal intraepithelial lesions.

It is interesting that with the two most commonly used antibodies CD31 and CD34, the highest MVD was seen in Ni Types IV and Va, the patterns typical for low-grade and high-grade dysplasia or preinvasive cancer and not in Type Vb or Vc, which are attributed to invasive cancer. The MVD decline with further progression of invasive cancer may be explained by the disarray and decomposition of vessels, as well as the accompanying necrosis. The variances in immunostaining results, when using different vascular markers, had been reported in previously published studies and are explained by varying expression of these factors, depending on the degree of maturation and differentiation of the vascular endothelial cells.^20^, ^21^ While CD31 is detected in mature endothelial cells, anti-CD34 antibodies also immunostain lymphatic endothelial cells, fibroblasts, adipocytes, what also may interfere with higher CD34 MVD count comparing to CD31 MVD.^21^ Another vascular marker worthy of attention, not analyzed in this study, is CD105 antibody that targets endoglin expressed throughout the process of vasculogenesis from immature to mature blood vessels.^21^

Only a few studies investigated and confirmed the correlation between the NBI vascular pattern classification and microvascular density. The study by Fujii et al. analyzed the microvascular irregularities assessed by NBI and CD34 MVD in pharyngeal cancer and revealed that architectural changes of IPCL corresponded with histopathologically- identified architectural disturbances and cellular atypia.^22^ Liu et al. presented a Spearman correlation coefficient of 0.67 between NBI and MVD CD34 expression in evaluating the angiogenesis of colorectal lesions.^12^

Kawamura et al. demonstrated a strong correlation between NBI and pathologically- assessed vascular density in gastric lesions.^23^ Moreover they also confirmed that NBI assessed differences in vascular density is reliable for distinguishing the differentiated and undifferentiated types of gastric carcinoma. Uno et al. proposed a capillary pattern classification for detecting dysplastic lesions in Barrett esophagus, and confirmed its applicability by not only demonstrating the accuracy of the classification but also by demonstrating significant differences in microvascular density between the classification types.^24^

## Conclusions

This study confirmed that the microvascular morphological changes of intraepithelial laryngeal lesions observed under NBI endoscopy are positively correlated with angiogenesis indexes of immunohistological evaluation, thus supporting the feasibility of endoscopy in the evaluation of angiogenesis.

## Ethical approval

This article does not contain any studies with animals performed by any of the authors. All procedures performed in studies involving human participants were in accordance with the ethical standards of the institutional and/or national research committee and with the 1964 Helsinki declaration and its later amendments or comparable ethical standards. Informed consent was obtained from all individual participants included in the study.

## Conflicts of interest

The authors declare no conflicts of interest.
